# Supplementary material for: A Combined Cell-Worm Approach to Search for Compounds Counteracting the Toxicity of Tau Oligomers In Vivo
Source: Int J Mol Sci. 2022 Sep 24;23(19):11277. doi: 10.3390/ijms231911277 (PMC9569484; doi:10.3390/ijms231911277)
Supplement: Supplementary file 1 [file ijms-23-11277-s001.zip › ijms-1859876-supplementary.pdf]

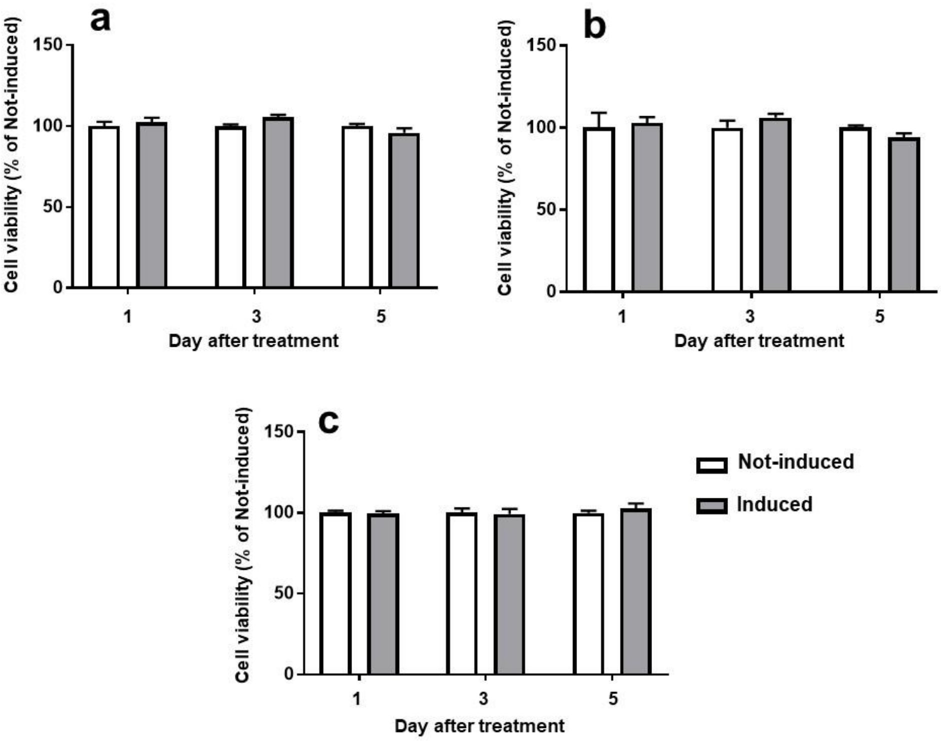

Figure S1. Tau P301L expression did not affect cell viability. HEK T-Rex cells were treated with 1.0 µg/mL doxycycline to induce the expression of human tau P301L (Induced) or with the same volume of 10 mM PBS, pH 7.4 (Not-induced). Cell viability was recorded 1, 3, and 5 days after treatment, with (**a**) MTT, (**b**) LDH, and (**c**) Alamar blue assays. Data are expressed as values normalized on the mean of Not-induced cells from three technical replicates of three independent experiments.

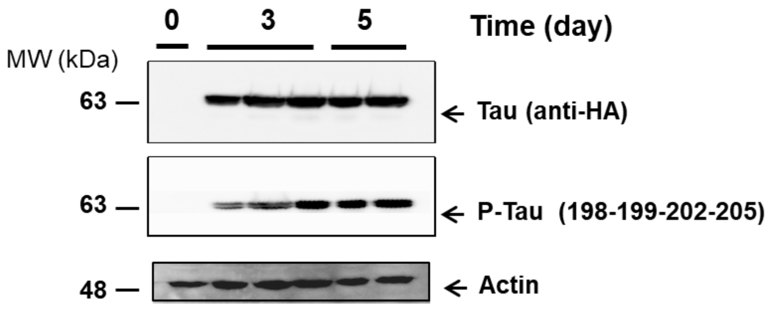

Figure S2. Tau P301L expression in HEK T-REx cells. Representative Western blot of total tau and phosphorylated tau (P-Tau) in lysates of HEK T-REx cells collected before (time 0) or 3 and 5 days after treatment with doxycycline. An equal amount of proteins were loaded in each gel lane (30 μg) and immunoblotted with an anti-HA tag, anti-P-tau (198-199-202-205), or anti-actin antibody.

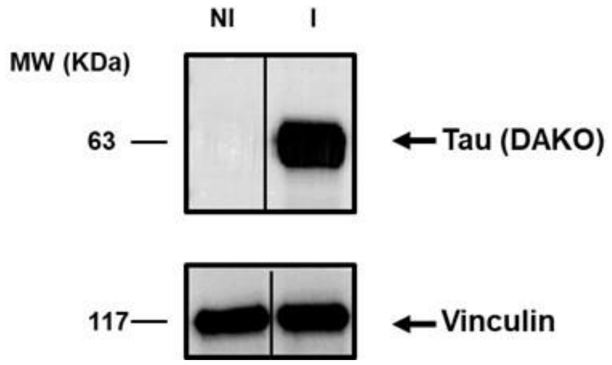

Figure S3. Western blot of cell lysates before immunoprecipitation and used for the input.
Cell lysates were prepared from HEK T-REx cells induced (I) or Not-induced (NI) for 5 days to express tau P301L. An equal amount of proteins (15 µg) was loaded in each lane of gel and immunoblotted with an anti-tau antibody (DAKO) or anti-vinculin antibody.

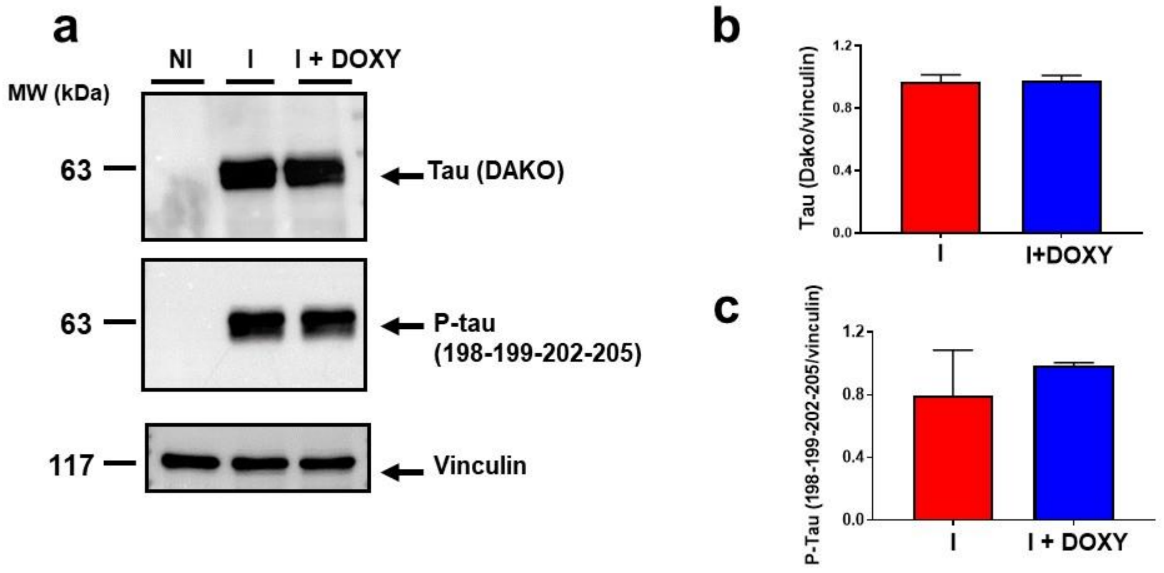

Figure S4. Effect of Doxy on tau expression. Representative Western blot of (**a**) total tau and phosphorylated tau (P-Tau) in lysates of HEK T-REx cells induced or not-induced (NI) for 5 days to express Tau$_{P301L}$. Cell lysates of Induced cells (30 μg) were incubated for 2 h with 50 μM Doxy (I+ DOXY) or the same volume of 10 mM PBS, pH 7.4 (I). Equal amounts of proteins (30 μg) were loaded in each gel lane and immunoblotted with **(a)** anti-tau antibody (DAKO), anti-P-tau (198-199-202-205) antibody, or anti-vinculin antibody. (**b**) Total tau and **(c)** P-tau quantification is expressed as the mean volume of the immunoreactive band/vinculin. Data are mean ± SD (N=3).
